# Pre- and perinatal hypoxia associated with hippocampus/amygdala volume in bipolar disorder

**DOI:** 10.1017/S0033291713001529

**Published:** 2013-06-27

**Authors:** U. K. Haukvik, T. McNeil, E. H. Lange, I. Melle, A. M. Dale, O. A. Andreassen, I. Agartz

**Affiliations:** 1 Department of Psychiatric Research, Diakonhjemmet Hospital, Oslo, Norway; 2 K. G. Jebsen Centre for Psychosis Research, Institute of Clinical Medicine, University of Oslo, Oslo, Norway; 3 Department of Psychiatric Epidemiology, Lund University, Lund, Sweden; 4 School of Psychiatry and Clinical Neurosciences, University of Western Australia, Perth, WA, Australia; 5 K. G. Jebsen Centre for Psychosis Research, Division of Mental Health and Addiction, Oslo University Hospital, Oslo, Norway; 6 Department of Neurosciences, University of California San Diego, La Jolla, CA, USA; 7 Department of Radiology, University of California San Diego, La Jolla, CA, USA

**Keywords:** MRI, neurodevelopment, neuroimaging, obstetric complications, psychosis

## Abstract

**Background:**

Pre- and perinatal adversities may increase the risk for schizophrenia and bipolar disorder. Hypoxia-related obstetric complications (OCs) are associated with brain anatomical abnormalities in schizophrenia, but their association with brain anatomy variation in bipolar disorder is unknown.

**Method:**

Magnetic resonance imaging brain scans, clinical examinations and data from the Medical Birth Registry of Norway were obtained for 219 adults, including 79 patients with a DSM-IV diagnosis of bipolar disorder (age 29.4 years, s.d. = 11.8 years, 39% male) and 140 healthy controls (age 30.8 years, s.d. = 12.0 years, 53% male). Severe hypoxia-related OCs throughout pregnancy/birth and perinatal asphyxia were each studied in relation to *a priori* selected brain volumes (hippocampus, lateral ventricles and amygdala, obtained with FreeSurfer), using linear regression models covarying for age, sex, medication use and intracranial volume. Multiple comparison adjustment was applied.

**Results:**

Perinatal asphyxia was associated with smaller left amygdala volume (*t* = −2.59, *p* = 0.012) in bipolar disorder patients, but not in healthy controls. Patients with psychotic bipolar disorder showed distinct associations between perinatal asphyxia and smaller left amygdala volume (*t* = −2.69, *p* = 0.010), whereas patients with non-psychotic bipolar disorder showed smaller right hippocampal volumes related to both perinatal asphyxia (*t* = −2.60, *p* = 0.015) and severe OCs (*t* = −3.25, *p* = 0.003). No associations between asphyxia or severe OCs and the lateral ventricles were found.

**Conclusions:**

Pre- and perinatal hypoxia-related OCs are related to brain morphometry in bipolar disorder in adulthood, with specific patterns in patients with psychotic *versus* non-psychotic illness.

## Introduction

The exact pathophysiology and aetiology of the severe mental disorders schizophrenia and bipolar disorder remain unknown. They have been hypothesized to be part of the same psychosis continuum, since they in addition to overlapping symptoms share some genetic underpinnings (Purcell *et al.* 2009), cognitive impairments (Simonsen *et al.* 2011) and brain anatomical abnormalities (Ellison-Wright & Bullmore, 2010). Whereas pre- and perinatal complications have been established as risk factors for schizophrenia (Fatemi & Folsom, 2009; Rapoport *et al.* 2012), the evidence for an association between pre- and perinatal adversities and the risk for bipolar disorder is less consistent. Some authors have argued that in genetically susceptible individuals, the absence of pre- and perinatal complications favours the development of bipolar disorder whereas their presence favours the development of schizophrenia (Murray *et al.* 2004; Demjaha *et al.* 2012).

Nevertheless, some epidemiological studies suggest that pre- and perinatal factors may increase the risk for bipolar disorder and affective psychosis. Hultman *et al.* (1999) have demonstrated an association between specific obstetric complications (OCs) and affective psychosis; an increasing birth weight was found to linearly associate with decreased risk for affective disorders (Abel *et al.* 2010); recently, increased risk for bipolar disorder in children born pre-term [odds ratio 2.7, 95% confidence interval (CI) 1.6–4.5] was reported (Nosarti *et al.* 2012). Accordingly, neurodevelopmental disturbances and/or pre- and perinatal trauma may also be of importance for the development of bipolar disorder.

Magnetic resonance imaging (MRI) studies have demonstrated the existence of neuroanatomical abnormalities in bipolar disorder (Hallahan *et al.* 2011), the most consistent finding being enlarged ventricular volumes (Kempton *et al.* 2008; Arnone *et al.* 2009; Rimol *et al.* 2010; Hallahan *et al.* 2011). The results for other brain structures differ among studies, possibly due to low sample sizes and confounding factors, such as lithium medication. Recent meta-analyses report that lithium-naive patients with bipolar disorder have smaller hippocampal and amygdala volumes as compared with patients who receive lithium medication and with healthy controls (Hallahan *et al.* 2011; Hajek *et al.* 2012). There is also some evidence supporting more pronounced brain abnormalities in patients with psychotic bipolar disorder than in patients with non-psychotic disorder (Strasser *et al.* 2005; Edmiston *et al.* 2011; Anticevic *et al.* 2013). The mechanisms underlying the structural brain abnormalities observed in bipolar disorder are not completely known. Post-mortem studies have demonstrated reduced neural somal size (Bezchlibnyk *et al.* 2007) and neuron numbers (Berretta *et al.* 2007) in the amygdala, and reduced number of parvalbumin- and somatostatin-expressing interneurons (Konradi *et al.* 2011) and reduced pyramidal cell size (Liu *et al.* 2007; Konradi *et al.* 2011) in the hippocampus of bipolar disorder patients. These neuronal changes may have a developmental origin, given the fact that animal models have demonstrated long-term neuronal loss in the amygdala (Carty *et al.* 2010) and reduced pyramidal cell size in the hippocampus (Rees *et al.* 2008) following pre- and perinatal hypoxia. Moreover, smaller hippocampal volumes and larger ventricular volumes have been demonstrated in schizophrenia patients with a history of pre- and perinatal hypoxia (Cannon *et al.* 2002; van Erp *et al.* 2002). Enlarged ventricles have also been observed in schizophrenia patients who suffered prolonged birth (indicating hypoxia) (McNeil *et al.* 2000). In schizophrenia, smaller hippocampal volumes have been reported following OCs in general (Ebner *et al.* 2008), and severe OCs have been reported to interact with the hypoxia-regulated *GRM3* (metabotropic glutamate receptor 3) gene to affect hippocampal volume (Haukvik *et al.* 2010). Smaller hippocampal volume and reduced grey matter have been observed in otherwise healthy subjects born very preterm (before week 33) (de Kieviet *et al.* 2012). Smaller hippocampal volume has also been reported in healthy adolescents following perinatal asphyxia (Maneru *et al.* 2003), and long-term reductions of the grey matter in the amygdala have been observed in children with neonatal hypoxia–ischaemia (Peterson *et al.* 2000). Hence, it is plausible that pre- and perinatal complications affect brain structure abnormalities associated with bipolar disorder.

The aim of the current study was to investigate the relationship between pre- and perinatal trauma and brain structure volumes in patients with bipolar disorder. Specifically, we studied the relationship between two measures of pre/perinatal trauma [i.e. an established composite severe OCs score comprising complications occurring throughout the whole pre- and perinatal period (McNeil *et al.* 1994), and a diagnosis of perinatal asphyxia, a distinct complication shown by animal models to cause long-term brain abnormalities (Rees *et al.* 2008; Carty *et al.* 2010)], and three brain volumes either previously reported to be associated with OCs in schizophrenia (i.e. hippocampus and lateral ventricles) or associated with bipolar disorder (i.e. amygdala).

We hypothesized that perinatal asphyxia and severe OCs would be associated with smaller hippocampus and amygdala volumes, and with larger ventricular volumes, in patients with bipolar disorder, and that the associations would be stronger in those with psychotic than in those with non-psychotic disorder. This latter prediction is based on the findings of more pronounced brain abnormalities (Strasser *et al.* 2005; Edmiston *et al.* 2011; Anticevic *et al.* 2013) and cognitive impairments (Simonsen *et al.* 2011) in the psychotic than non-psychotic form of bipolar disorder. In addition, psychotic bipolar disorder may be more similar to schizophrenia, a disorder in which patients with OCs show more pronounced brain abnormalities than patients without such complications (McNeil *et al.* 2000; van Erp *et al.* 2002; Schulze *et al.* 2003, Ebner *et al.* 2008).

To our knowledge, this is the first study to explore the association between hypoxia-related OCs, in particular perinatal asphyxia, and neuroanatomy in bipolar disorder.

## Method

### Subjects

The subject sample (*n* = 219) consisted of patients with a Diagnostic and Statistical Manual of Mental Disorders, Fourth Edition (DSM-IV) diagnosis within the bipolar spectrum (*n* = 79) – bipolar I disorder (DSM-IV 296.0-7) (*n* = 47), bipolar II disorder (DSM-IV 296.89) (*n* = 28), or bipolar disorder *not otherwise specified* (DSM-IV 296.80) (*n* = 4) – and healthy controls (*n* = 140) from the ongoing multi-centre Thematically Organized Psychosis Study at the University of Oslo, Norway. All subjects were born in Norway and aged between 18 and 42 years at the start of the study.

Patients were included from four major psychiatric hospitals and their out-patient clinics that together cover most of the population in Oslo. All patients underwent thorough clinical investigation by specially trained psychologists and physicians. Clinical diagnoses were assessed using the Structured Clinical Interview for DSM-IV Axis I disorders (SCID-I) module A–E (Spitzer *et al.* 1988), with an overall agreement for diagnostic categories of 82% (*κ* = 0.77, 95% CI 0.60–0.94). Psychosocial function was assessed with the Global Assessment of Function scale – split version. Affective state was assessed with the Young Mania Rating Scale and the *Inventory of Depressive Symptomatology* (IDS) scale. Current psychotic symptoms were rated by the use of the Positive and Negative Syndrome Scale (PANSS), with intraclass coefficients of 0.73 and 0.86 for the positive and negative subscales, respectively (Engh *et al.* 2010). Patients were classified as having psychotic (*n* = 48) or non-psychotic (*n* = 31) bipolar disorder based on the presence of current psychotic episode (defined as a score of 4 or above on any one of the PANSS items P1, P3, P5, P6, G9), or a history of psychosis based on information retrieved from the SCID-I interview.

The healthy control subjects were randomly selected from the national population register, and resident in the same catchment area as the patients. They were interviewed for symptoms of severe mental illness by trained psychologists and examined with the Primary Care Evaluation of Mental Disorders (Spitzer *et al.* 1994) to ensure no current or previous psychiatric disorders. Control subjects with current or previous somatic illness, or substance misuse disorder including alcohol overuse that could affect brain morphology were excluded. Demographic and clinical variables are listed in Table 1.

**Table 1.** Demographic, clinical and birth-related characteristics

	Psychotic BD (*n* = 48)		Non-psychotic BD (*n* = 31)		Control subjects (*n* = 140)		*p* ^a^
	Mean (s.d.)	Range	Mean (s.d.)	Range	Mean (s.d.)	Range	
Sex, *n* (%)							0.024
Male	23 (48)		8 (26)		74 (53)		
Female	25 (52)		23 (74)		66 (47)		
Handedness, *n* (%)							0.660
Right	39 (87)		28 (90)		130 (93)		
Left	5 (11)		3 (10)		9 (6)		
Ambidextrous	1 (2)		0 (0)		1 (1)		
Perinatal asphyxia, *n* (%)							0.314
Yes	9 (19)		7 (23)		18 (13)		
No	39 (81)		24 (77)		122 (87)		
Severe OCs, *n* (%)							0.209
Yes	18 (37)		9 (29)		34 (24)		
No	30 (63)		22 (71)		106 (76)		
Age, years	29.9 (6.7)	19–42	28.7 (4.8)	18–42	30.8 (6.1)	18–42	0.200
Gestational age, weeks	39.7 (2.4)	31–44	40.2 (1.9)	35–43	39.9 (1.8)	35–45	0.535
Maternal age, years	28.0 (4.7)	18–39	27.7 (5.8)	20–40	27.3 (4.8)	18–40	0.690
Birth weight, g	3570 (623)	1880–4900	3523 (456)	2580–4560	3518 (528)	2200–4990	0.843
Birth head circumference, cm (*n* = 103)	35.4 (1.4)	32–38	34.8 (1.5)	32–38	35.3 (1.4)	32–38	0.438
Duration of education, years	13.6 (2.3)	9–20	13.6 (2.2)	9–18	14.1 (2.2)	9–20	0.229
NART IQ	106.2 (4.1)	97.6–114.2	106.6 (3.2)	98.7–112.2	107.0 (3.7)	97.7–114.7	0.094
YMRS	2.4 (3.1)	0–11	2.4 (2.8)	0–8			0.980
IDS	15.6 (13.0)	0–50	22.6 (11.6)	7–51			0.021
GAF symptom	57 (11)	35–80	57 (7)	42–69			0.951
GAF function	53 (14)	31–82	59 (9)	43–75			0.063
PANSS positive	10.2 (3.6)	7–25	9.8 (2.8)	7–17			0.775
PANSS negative	10.7 (4.0)	7–25	10.6 (3.7)	7–21			0.867
Age at illness onset, years	25.9 (7.2)	14–41	24.7 (5.2)	16–38			0.408
Medication, DDD (*n* = pBD/npBD)							
Antipsychotics (*n* = 30/4)	0.9 (0.6)	0.1–2.5	0.6 (0.3)	0.5–1.0			0.358
Antiepileptics (*n* = 21/17)	0.8 (0.4)	0.7–1.3	0.5(0.3)	0.3–1.1			0.064
Antidepressants (*n* = 20/13)	1.5 (0.8)	0.3–3.0	1.6 (0.7)	0.8–3.0			0.794
Lithium (*n* = 8/4)	1.1 (0.4)	0.5–1.8	0.9 (0.3)	0.5–1.3			0.587

BD, Bipolar disorder; s.d., standard deviation; OCs, obstetric complications as scored by the McNeil Sjöström scale; NART IQ, NART National Adult Reading Test; YMRS Young Mania Rating Scale; IDS, Inventory of Depressive Symptomatology; GAF, Global Assessment of Function scale – split version; PANSS, Positive and Negative Syndrome Scale; DDD, defined daily dosage; pBD psychotic BD; npBD non-psychotic BD.

Data are given as number (percentage) or as mean (s.d.) and range.

a Analysed by *χ*^2^ for sex, handedness, perinatal asphyxia and severe OCs; otherwise analysis of variance.

The study was approved by the Regional Committee for Medical Research Ethics and the Norwegian Data Inspectorate, and was conducted in accordance with the Helsinki declaration. After complete description of the study to the subjects, written informed consent was obtained from all participating subjects.

### Birth data

The birth data were collected from the Medical Birth Registry of Norway (MBRN). In Norway, all births after gestational week 16 are compulsorily reported to the MBRN by the attending midwife or physician (Irgens, 2000). Medical and sociodemographic data pertaining to the pregnancy, birth, and the health of the mother and the newborn are recorded on a standardized notification form by the midwife or doctor who attend the birth and reported to the MBRN. This notification form has remained unchanged during the period in which all subjects in the current study were born (1967–1991). Based on the information from the MBRN, i.e. birth data from the mother and the health of the newborn (the patient), one physician (U.K.H.) who was blinded to patient/control status scored presence and severity of OCs according to the McNeil–Sjöström scale (McNeil & Sjöström, 1995). The validated McNeil–Sjöström scale (McNeil *et al.* 1994) includes several hundred items of potential harm to the fetus, each classified according to severity on an ordinal scale from 1 to 6. In the present study, severe OCs were considered present in subjects who had experienced one or more complications of grade 5 or 6, whereas subjects with complications of grade 4 and below were classified as not having had severe OCs. A complication of grade 5 is defined as ‘potentially clearly greatly relevant/harmful’ (e.g. severe pre-eclampsia or perinatal asphyxia), and a complication of grade 6 is defined as a complication that causes ‘very great harm to or deviation in offspring’ (e.g. eclampsia, offspring hypoxic–ischaemic cerebral injury). In addition, a diagnosis of perinatal asphyxia from the MBRN was recorded separately, to constitute a narrower hypoxic OCs parameter. By definition, all subjects recorded to have a diagnosis of asphyxia are classified as having severe OCs according to the McNeil–Sjöström scale. Birth-related variables are listed in Table 1.

### MRI acquisition

All participants underwent MRI scanning on a 1.5 T Siemens Magnetom Sonata scanner (Siemens Medical Solutions, Germany) equipped with a standard head coil. Two sagittal T1-weighted magnetization-prepared rapid gradient echo volumes were acquired with the Siemens tfl3d1_ns pulse sequence (echo time = 3.93 ms, repetition time = 2730 ms, inversion time = 1000 ms, flip angle = 7°, field of view = 24 cm, voxel size = 1.33  ×  0.94  ×  1 mm^3^, number of partitions = 160) and subsequently averaged together, after rigid-body registration, to increase the signal to noise ratio. There was no scanner upgrade during the study period, and patients and controls were scanned consecutively. A neuroradiologist evaluated all scans, and scans with brain pathol ogy were excluded.

### MRI post-processing

FreeSurfer software (version 4.5.0) (http://surfer.nmr.mgh.harvard.edu) was used to obtain hippocampal, amygdala, ventricular and intracranial volumes (ICVs) from T1-weighted images (Fischl, 2012). Briefly, the subcortical segmentation algorithm combines information on image intensity, probabilistic atlas location, and the local spatial relationships between structures to automatically assign a neuroanatomical label to each voxel in the MRI volume (Fischl *et al.* 2002). The reliability of the automatic volume measurements has been tested against manual tracing, and the agreement between the automatically obtained volumes and manual tracings was comparable with the agreement between manual tracings obtained by two different experts (Fischl *et al.* 2002). The procedures were fully automated without manual editing. All scans were visually inspected following standard procedures.

### Statistical analyses

All statistical analyses were performed within the statistical package SPSS version 20 (IBM SPSS Inc., USA). Group differences in demographic, clinical, obstetric and neurocognitive variables were analysed with Student's *t* test and *χ*^2^ statistics. All statistical analyses were two-tailed.

Diagnostic differences in brain volumes were assessed with a general linear model with the brain structure of interest as the dependent variable, diagnosis (psychotic bipolar disorder, non-psychotic bipolar disorder and healthy controls) as a fixed factor, and age, sex, ICVs and lithium use as covariates.

Hierarchical linear regression forced-entry models were used to study the associations between pre- and perinatal complications and brain structure within each diagnostic group, with the brain structure of interest as the dependent variable, and age, sex, and ICVs as independent variables entered in the first block, and perinatal asphyxia or severe OCs (both as dichotomous variables) in the second block. To adjust for multiple tests of brain volumes, Bonferroni correction was applied. Since the left and right hemisphere volumes were highly correlated (Pearson correlation, *r* = 0.84, *p* < 0.0001 for hippocampus; *r* = 0.80, *p* < 0.0001 for amygdala; *r* = 0.84, *p* < 0.0001 for the lateral ventricles), and the severe OCs/perinatal asphyxia categories were highly overlapping (all subjects with perinatal asphyxia were also classified as having severe OCs), the corrected threshold for statistical significance was set to 0.017 [0.05/3 brain structures (hippocampus, amygdala and lateral ventricles)]. Because lithium use is known to affect hippocampal and amygdala volumes (Hallahan *et al.* 2011), defined daily dosages (DDDs) of lithium were calculated according to the guidelines from the World Health Organization Collaborating Center for Drug Statistics Methodology (http://www.whocc.no/atcdd), and controlled for in the statistical analyses. DDDs were also calculated for antipsychotic medication, and preliminary analyses showed no association between either DDD or generation of antipsychotic medication to hippocampal/amygdala/ventricular volumes. Moreover, including handedness as a covariate in the statistical model did not affect the results. Hence, handedness, and DDD and generation of antipsychotic medication were not included in the main statistical analyses.

## Results

### Demographic, clinical and birth-related variables

Demographic, clinical and birth-related characteristics are given in Table 1. There were significantly more women in the non-psychotic bipolar disorder group [*χ*^2^ = 7.74, degrees of freedom (df) = 2, *p* = 0.024] and patients with non-psychotic bipolar disorder had significantly more current depressive symptoms (higher scores on the IDS) than patients with psychotic illness (*F* = 5.54, df = 1, *p* = 0.021). No other significant differences between groups were found. Noteworthy, patients with bipolar disorder and healthy controls did not differ in the prevalence of perinatal asphyxia (*χ*^2^ = 2.11, df = 1, *p* = 0.147) or severe OCs (*χ*^2^ = 2.46, df = 1, *p* = 0.117). Within the bipolar group, subjects with psychotic and non-psychotic illness did not differ in the prevalence of perinatal asphyxia (*χ*^2^ = 0.171, df = 1, *p* = 0.679) or severe OCs (*χ*^2^ = 0.600, df = 1, *p* = 0.438).

### Case–control differences in brain volumes

Ventricular, hippocampal and amygdala volumes did not differ between patients with psychotic bipolar disorder, non-psychotic bipolar disorder and healthy controls, when age, sex, ICV and lithium use were controlled for (Table 2). Nor were there any significant differences in brain volumes between the whole bipolar disorder patient group (patients with psychotic and non-psychotic bipolar disorder combined) and healthy controls (*F* = 1.50, *p =* 0.221 for the left and *F* = 1.50, *p* = 0.222 for the right hippocampus; *F* = 0.75, *p* = 0.388 for the left and *F* = 0.41, *p* = 0.525 for the right amygdala; *F* = 1.33, *p* = 0.249 for the left and *F* = 1.06, *p* = 0.304 for the right lateral ventricles).

**Table 2.** Brain structure volumes (in mm^2^) in BD patients and healthy controls

	Psychotic BD	Non-psychotic BD	Controls	Statistics^a^
Amygdala				
Left	1722 (210)	1641 (163)	1695 (223)	*F* = 0.69, *p* = 0.693
Right	1736 (217)	1670 (191)	1718 (241)	*F* = 0.43, *p* = 0.958
Hippocampus				
Left	4105 (453)	3934 (384)	4150 (391)	*F* = 1.29, *p* = 0.278
Right	4195 (414)	4064 (386)	4259 (419)	*F* = 1.09, *p* = 0.339
Lateral ventricles				
Left	8083 (4649)	7243 (4625)	7364 (3833)	*F* = 1.42, *p* = 0.245
Right	7463 (4548)	6756 (4500)	6924 (3542)	*F* = 1.36, *p* = 0.260

BD, Bipolar disorder.

Data are given as mean (standard deviation).

a General linear model, diagnostic differences when age, sex, intracranial volume and lithium use are controlled for.

### Associations between perinatal asphyxia, severe OCs and brain volumes

Perinatal asphyxia was associated with smaller left amygdala volume (*t*_75_ = −2.57, *p* = 0.012) in bipolar disorder patients, but not in healthy controls (*t*_136_ = 1.46, *p* = 0.145), when age, sex and ICV were controlled for (Table 3). Including lithium use, gestational age and birth weight in the statistical model did not affect the results substantially, with asphyxia still being associated with smaller left amygdala volume (*t*_74_ = −2.56, *p* = 0.012; *t*_74_ = −2.15, *p* = 0.035; and *t*_74_ = −2.56, *p* = 0.013, including lithium use, gestational age and birth weight, respectively). Severe hypoxia-related OCs were associated with smaller right hippocampal volume (*t*_75_ = −2.25, *p* = 0.029) in bipolar disorder patients but not in healthy controls (*t*_136_ = 0.223, *p* = 0.824), an association that was stronger when lithium use was included in the model (*t*_74_ = −2.40, *p* = 0.019). This association did not remain significant after Bonferroni correction for multiple comparisons. No associations between asphyxia or severe OCs and the lateral ventricles were found in either patients with bipolar disorder or healthy control subjects.

**Table 3.** Associations between severe OCs, perinatal asphyxia and brain structure volumes in patients with bipolar disorder (n = 79) and healthy controls (n = 140)^a^

	Severe OCs					Perinatal asphyxia				
	*t*	*p*	adj *p*	*B*	(s.e.)	*t*	*p*	adj *p*	*B*	(s.e.)
Left hippocampus										
Bipolar	−0.99	0.324	0.972	−93.3	(94.0)	−1.00	0.319	0.957	−114.6	(114.2)
Controls	−0.17	0.866	1	−10.8	(64.0)	0.15	0.884	1	11.9	(81.8)
Right hippocampus										
Bipolar	−2.23	0.029	0.087	−186.7	(83.9)	−1.59	0.117	0.351	−164.3	(103.6)
Controls	0.22	0.824	1	15.6	(70.0)	−0.09	0.929	1	−7.9	(89.3)
Left amygdala										
Bipolar	−1.84	0.069	0.207	−76.0	(41.2)	−2.57	0.012	0.036	−126.1	(49.1)
Controls	1.36	0.177	0.531	49.9	(36.8)	1.46	0.145	0.435	68.9	(47.0)
Right amygdala										
Bipolar	−0.65	0.521	1	−30.7	(47.6)	−1.36	0.180	0.540	−77.7	(57.3)
Controls	0.05	0.963	1	1.9	(41.2)	0.90	0.372	1	47.1	(52.5)
Left lateral ventricle										
Bipolar	−0.48	0.631	1	−743.8	(1012.5)	−0.33	0.744	1	−404.6	(1234.1)
Controls	1.23	0.221	0.663	907.3	(710.5)	0.22	0.842	1	204.0	(914.0)
Right lateral ventricle										
Bipolar	0.56	0.574	1	315.5	(973.9)	0.55	0.581	1	655.1	(1182.0)
Controls	1.18	0.242	0.726	975.6	(644.2)	0.82	0.414	1	679.2	(828.8)

Severe OCs, Severe hypoxia-related obstetric complications; adj *p*, adjusted *p* values after Bonferroni correction; s.e., standard error.

a Linear regression analyses controlled for effects of age, sex and intracranial volume within each diagnostic group.

When splitting the patients with bipolar disorder into patients with psychotic and non-psychotic disorder, patients with psychotic bipolar disorder showed distinct associations between perinatal asphyxia and smaller left amygdala volume (*t*_44_ = −2.69, *p* = 0.010), whereas patients with non-psychotic bipolar disorder showed smaller right hippocampal volumes related to both perinatal asphyxia (*t*_28_ = −2.60, *p* = 0.015) and severe OCs (*t*_28_ = −3.25, *p* = 0.003) (Table 4). Hence, the amygdala findings in the whole group of patients with bipolar disorder appear to be driven by the patients with psychotic bipolar disorder, and the hippocampus findings by the patients with non-psychotic bipolar disorder. When lithium use, gestational age and birth weight were included in the statistical model, perinatal asphyxia was still associated with smaller left amygdala volume in patients with psychotic bipolar disorder (*t*_43_ = −2.62, *p* = 0.012; *t*_43_=−2.47, *p* = 0.018; and *t*_43_ = −2.6, *p* = 0.011, including lithium use, gestational age and birth weight, respectively). In the non-psychotic bipolar disorder group, the association between perinatal asphyxia and smaller right hippocampus volume remained significant when DDDs of lithium use were included in the model (*t*_27_=−2.62, *p* = 0.015), and was nominally significant when gestational age (*t*_27_ = −2.15, *p* = 0.042) and birth weight (*t*_27_ = −2.44, *p* = 0.022) were included in the model. The association between smaller right hippocampus volume and severe OCs also remained significant when DDDs of lithium use were included in the model (*t*_27_ = −3.20, *p* = 0.004). Worth noting, the associations between perinatal asphyxia/severe OCs and amygdala/hippocampus volume had the same direction in the opposite hemisphere structures, but these associations did not reach statistical significance (Table 4).

**Table 4.** Associations between severe OCs, perinatal asphyxia and brain structure volumes in patients with psychotic (n = 48) and non-psychotic (n = 31) BD^a^

	Severe OCs					Perinatal asphyxia				
	*t*	*p*	adj *p*	*B*	(s.e.)	*t*	*p*	adj *p*	*B*	(s.e.)
Left hippocampus										
Non-psychotic BD	−2.49	0.019	0.057	−386.2	(155.0)	−1.81	0.082	0.246	−333.2	(178.0)
Psychotic BD	−0.09	0.93	1	−11.0	(124.6)	−0.08	0.936	1	−12.3	(153.2)
Right hippocampus										
Non-psychotic BD	−3.25	0.003	0.009	−485.6	(149.5)	−2.60	0.015	0.045	−449.0	(173.0)
Psychotic BD	−0.77	0.445	1	−82.4	(106.8)	0.06	0.95	1	8.3	(132.2)
Left amygdala										
Non-psychotic BD	−1.19	0.244	0.732	−85.6	(71.8)	−0.94	0.359	1	−74.2	(79.4)
Psychotic BD	−1.95	0.058	0.168	−107.3	(55.1)	−2.69	0.010	0.030	−175.5	(65.4)
Right amygdala										
Non-psychotic BD	−0.27	0.791	1	−22.6	(83.0)	0.30	0.764	1	27.5	(90.8)
Psychotic BD	−1.15	0.257	0.771	−72.8	(63.4)	−2.26	0.029	0.087	−168.8	(74.9)
Left lateral ventricle										
Non-psychotic BD	0.23	0.871	1	389.3	(1162.1)	−0.29	0.772	1	−531.5	(1818.8)
Psychotic BD	−1.65	0.107	0.321	−2172.7	(1319.6)	−0.76	0.442	1	−1288.1	(1661.5)
Right lateral ventricle										
Non-psychotic BD	0.90	0.377	1	1303.2	(1450.6)	0.52	0.610	1	827.9	(1604.6)
Psychotic BD	−0.77	0.447	1	−986.2	(1286.2)	−0.29	0.776	1	−456.3	(1590.9)

Severe OCs, Severe hypoxia-related obstetric complications; BD, bipolar disorder; adj *p*, adjusted *p* values after Bonferroni correction; s.e., standard error.

a Linear regression analyses controlled for effects of age, sex and intracranial volume within each diagnostic group.

## Discussion

We found that perinatal asphyxia and severe OCs were related to smaller amygdala and hippocampal volume in patients with bipolar disorder. Whereas patients with psychotic bipolar disorder showed reduced amygdala volume following perinatal asphyxia, patients with non-psychotic bipolar disorder showed reduced hippocampal volume following perinatal asphyxia and severe OCs, after adjustment for multiple comparisons, and controlling for the effects of age, sex, ICV and medication use. To the best of our knowledge, this is the first study to investigate associations between hypoxia-related pre- and perinatal complications and brain MRI morphometry in bipolar disorder.

Our findings indicate that perinatal hypoxic brain trauma is of importance for the adult brain morphology in bipolar disorder, and may thus be a neurodevelopmental factor of importance to disease development. This concurs to some extent with large-scale epidemiological studies that report lower birth weight (Abel *et al.* 2010), specific OCs (Hultman *et al.* 1999) and premature birth (Nosarti *et al.* 2012) to increase the risk for bipolar disorder or affective psychosis. Indeed, we have in a subject sample overlapping with the current study previously demonstrated lower birth weight to correlate with smaller brain cortical surface area in patients across the psychosis spectrum as well as healthy controls (Haukvik *et al.* 2013). The results from the current study expand on this by demonstrating distinct associations between specific hypoxia-related pre- and perinatal complications and subcortical structures, known to be vulnerable to perinatal hypoxia (Carty *et al.* 2010; Morales *et al.* 2011), in patients with bipolar disorder. As such, the current findings to some extent support the speculation by Nosarti *et al.* (2012) that there may exist a neurodevelopmental subtype of bipolar disorder.

Within the whole group of bipolar disorder patients, we found perinatal asphyxia to be significantly associated with smaller left amygdala volume. The amygdala is involved in emotion processing and regulation, disturbances of which are core features of bipolar disorder (Townsend & Altshuler, 2012). Altered amygdala function related to emotion-processing tasks has repeatedly been reported from functional MRI studies in patients with bipolar disorder (Cerullo *et al.* 2009). Emotional dysregulation and impaired stress response, other important features of bipolar disorder (Brietzke *et al.* 2012), may be caused by disturbances in corticotropin metabolism and dysfunction in the hypothalamic–pituitary–adrenal (HPA) axis (Gilmor *et al.* 2003; Spijker *et al.* 2009). Interestingly, a recent rat model study demonstrated significant long-term loss, shrinkage of cell soma size, and axonal degeneration of corticotropin-releasing factor-positive neurons in the amygdala following neonatal hypoxia–ischaemia (Carty *et al.* 2010). These changes were associated with increased locomotor activity and exploratory behaviour (Carty *et al.* 2010), behavioural abnormalities that are also observed in patients with bipolar disorder (Minassian *et al.* 2010; Perry *et al.* 2010). Moreover, increased anxiety has been reported following perinatal asphyxia and is associated with dopamine-innervated neurocircuitries in the amygdala, among other structures (Morales *et al.* 2011). Dopaminergic pathways are particularly vulnerable to perinatal asphyxia (Morales *et al.* 2011), and are also involved in the pathophysiology of psychotic disorders (Howes *et al.* 2009). Taken together, it seems biologically plausible that perinatal asphyxia is associated with long-term alterations in the structure of the amygdala, as our results suggest. Such alterations may be functionally associated with the distinct behavioural abnormalities observed in bipolar disorder.

Instead of confirming our initial hypothesis that the associations between perinatal asphyxia/severe OCs would be stronger in patients with psychotic than non-psychotic bipolar disorder, the results indicate different patterns of associations in psychotic *versus* non-psychotic bipolar disorder. Within a psychosis continuum, psychotic bipolar disorder would be considered to be closer than non-psychotic bipolar disorder to schizophrenia. In schizophrenia, smaller hippocampal volumes have been associated with pre- and perinatal trauma (van Erp *et al.* 2002; Schulze *et al.* 2003; Ebner *et al.* 2008). Surprisingly, we found no associations between severe OCs or perinatal asphyxia and smaller hippocampal volume in patients with psychotic bipolar disorder, but we did find such associations in patients with non-psychotic bipolar disorder. The biological validity of this association is, nevertheless, supported by the literature. First, animal models have demonstrated the pyramidal neurons within the hippocampus to be sensitive to prenatal hypoxia (Rees *et al.* 2008). Second, in the human neonate, hippocampal neurocircuitries are reported to be particularly vulnerable to hypoxia (Morales *et al.* 2011), and, third, healthy adolescents who have suffered perinatal asphyxia exhibit reduced hippocampal volumes (Maneru *et al.* 2003). Based on these findings, one could expect to find associations between perinatal hypoxia or severe OCs and hippocampal volume in the healthy controls as well, as we did in a previous study of schizophrenia patients and healthy controls (Haukvik *et al.* 2010). The hippocampus is, however, one of the brain regions in which neurogenesis occurs (Curtis *et al.* 2011), and the number of hippocampal neurons (Curlik & Shors, 2013), as well as the hippocampal volume as measured by MRI (Pajonk *et al.* 2010), might increase in response to different training tasks. On the other hand, hippocampal volume may be reduced in alcohol dependence (Agartz *et al.* 1999; De Bellis *et al.* 2000) and heavy cannabis use (Cousijn *et al.* 2012), as well as in several mental disorders including schizophrenia (Adriano *et al.* 2012; Shepherd *et al.* 2012), unipolar depression (Arnone *et al.* 2012) and bipolar disorder (Hallahan *et al.* 2011; Hajek *et al.* 2012), although we did not confirm the latter in this study. As such, a variety of factors may confound and interfere with putative associations between pre- and perinatal trauma and adult hippocampal volume.

The number of possible known and unknown confounders not accounted for constitute one limitation in the current study. Although we did control for current lithium use, which is known to affect hippocampal and amygdala volumes in bipolar disorder, we did not have reliable data on cumulative medication use. Another possible limitation to the generalizability of the current study is the inclusion of patients across mood states, i.e. depression, mania/hypomania, and euthymia, since it has been suggested that amygdala volume may fluctuate across mood states (Foland-Ross *et al.* 2012). Third, the subject groups were relatively small when the bipolar disorder group was split into psychotic and non-psychotic subgroups, which may have caused type II errors within the statistical analyses. Finally, by studying severe OCs, and comparing subjects with them *versus* all other subjects (including those with less severe OCs), we placed high demands on the strength of the relationship, and may have missed possible associations between brain structure and less severe OCs. Strengths of the current study include the use of unbiased birth registry data, thorough clinical characterization of participating subjects, and the use of one MRI scanner with no upgrades during the study period.

In summary, we report perinatal asphyxia to be related to smaller amygdala volume in patients with bipolar disorder. This suggests a neurodevelopmental component in the brain morphology of bipolar disorder. The different associations between pre- and perinatal complications and brain morphology observed in patients with psychotic and non-psychotic bipolar disorder, as well as their possible functional consequences, warrant further investigation.
